# A transcriptomic (RNA-seq) analysis of Drosophila melanogaster adult testes overexpressing microRNA-2b-1

**DOI:** 10.1016/j.dib.2022.108748

**Published:** 2022-11-13

**Authors:** Sharvin Manickam, Shallinie Thangadurai, Azali Azlan, Zarina Amin, Ghows Azzam, Mardani Abdul Halim

**Affiliations:** a School of Biological Sciences, Universiti Sains Malaysia, Penang 11800, Malaysia; b Malaysia Genome and Vaccine Institute, National Institutes of Biotechnology Malaysia, Jalan Bangi, Kajang, Selangor 43000, Malaysia; c Biotechnology Research Institute, Universiti Malaysia Sabah, Jalan UMS, Kota Kinabalu, Sabah 88400, Malaysia

**Keywords:** RNA-seq, Transcriptomics, Drosophila melanogaster, Testicular bulging, Testes, miR-2b-1, miRNAs

## Abstract

MicroRNAs (miRNAs) are short non-coding single-stranded RNAs with approximately 22 nucleotides in length that negatively regulate the mRNA translation of a target gene. MiR-2b-1 belongs to the largest miR-2 family in *Drosophila melanogaster* with 8 members and this miRNA family is conserved in invertebrates. miRNAs play key roles in gene regulation, cell proliferation, cell death, cell differentiation and cell developmental homeostasis in multicellular organisms. Its role in various human diseases is continuously being studied. miRNAs also found out to be crucial in maintaining stem cell niche in *D. melanogaster* gonads. We have identified that ectopic overexpression of miR-2b-1 of *D. melanogaster* causes testicular bulging (a tumour like phenotype) in 3-5 days old adult flies. Hence, we have performed a transcriptomic (RNA-seq) analysis to understand the role of miR-2b-1 in the development, maintenance, and differentiation of *D. melanogaster* adult testis stem cells. Data are available from GEO (accession number GSE211399).

**Specifications Table**

**Table:** 

Subject	Omics: Transcriptomics
Specific subject area	Developmental biology, *Drosophila melanogaster* genetics
Type of data	RNA-Seq raw data Processed data containing genes and their expression levels
How the data were acquired	Total RNA was extracted from miR-2b-1 overexpressing adult bulged testes using Qiagen RNeasy Mini Kit. Sequencing performed using Illumina HiSeq^TM^ platform. Galaxy bioinformatics tool was used to analyze the data.
Data format	Raw: Fastq.gz Analyzed: tabular
Description of data collection	Total RNA was extracted from 3-5 days old adult fly control testes and testes with miR-2b-1 overexpression using Trizol and Qiagen RNeasy mini kit. These samples were sequenced, and transcriptome were analyzed.
Data source location	Institution: School of Biological Sciences, Universiti Sains Malaysia City/Town/Region: Gelugor Country: Malaysia Latitude and longitude (and GPS coordinates, if possible) for collected samples/data: 5.3557° N, 100.3012° E
Data accessibility	Repository name: Gene Expression Omnibus (GEO) Data identification number: GSE211399 Direct URL to data: https://www.ncbi.nlm.nih.gov/geo/query/acc.cgi?acc=GSE211399

**Value of the Data**

• Various studies have shown the role of miRNAs in the development and maintenance of stem cells in *Drosophila* testes. However, this data is the first to report the transcriptomic analysis of testicular bulging in *Drosophila melanogaster* upon an ectopic overexpression of a miRNA.
• This analysis could provide a comprehensive overview of the role of miR-2b-1 in the development of *D. melanogaster* testicular stem cell niche.
• This data could shed light on the crucial role of miRNAs in regulating the genes that are crucial for the stem cell development, maintenance, and differentiation.
• The identification of pathways that are regulated by the miR-2b-1, could assist a better understanding of the role of the largest miRNA family (miR-2) in the development of *D. melanogaster*.

## 1 Objective

This data was analyzed to look at the genes and biological processes that are differentially regulated in miR-2b-1 overexpressing bulged testes.

## 2 Data Description

**Fig. 1:**
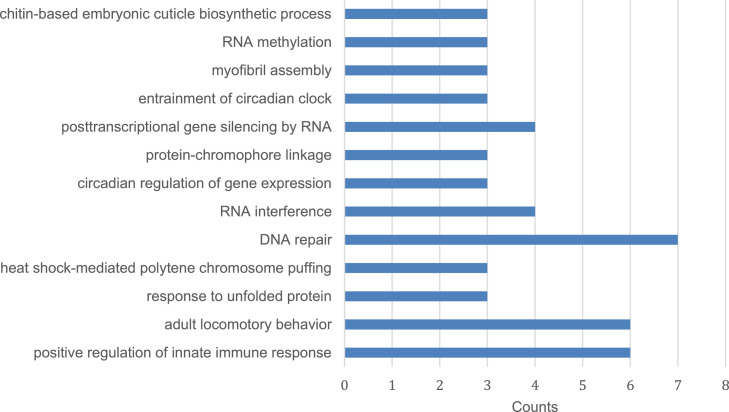
Enriched GO terms of biological processes for 439 differentially expressed genes. The highest gene count was observed under DNA repair category followed by adult locomotory behavior and positive regulation of innate immune response and post-transcriptional gene silencing by RNA and RNA interference round up the top 3 gene count. Significantly expressed gene counts (*P*-value<0.05) categorized under biological processes.

**Fig. 2:**
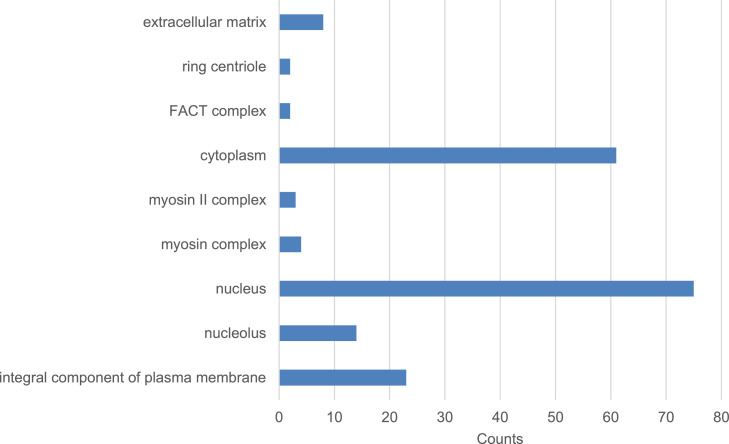
Enriched GO terms of cellular components for 439 differentially expressed genes. The top 3 of the highest gene count was observed under nucleus category followed by cytoplasm and integral component of plasma membrane. Significantly expressed gene counts (*P*-value<0.05) categorized under cellular components.

**Fig. 3:**
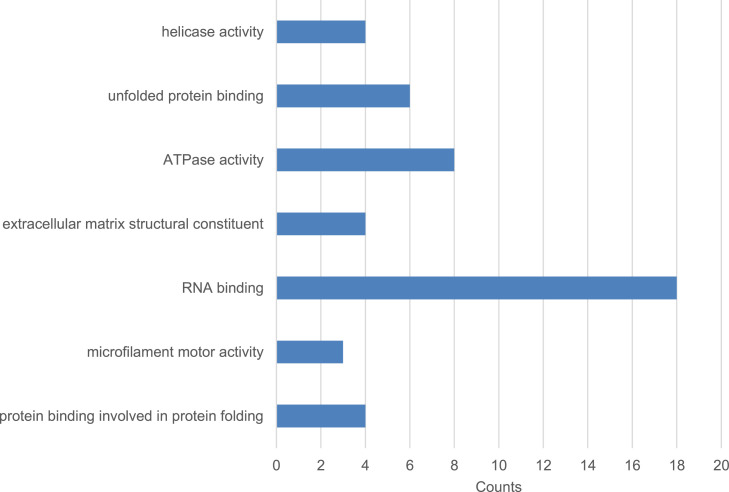
Enriched GO terms of molecular functions for 493 differentially expressed genes. Top 3 of the highest gene count was observed under RNA-binding process followed by ATPase activity and finally unfolded protein binding process. Significantly expressed gene counts (*P*-value<0.05) categorized under molecular functions.

Micro RNAs (miRNAs) are short, (approximately 22 nt long) non-coding RNAs that are endogenously regulates genes that are required for various developmental processes [1]. mir-2 is the largest miRNA family in *D. melanogaster* with 8 members; *mir-2a-1, mir-2a-2, mir-2b-1, mir-2b-2, mir-2c, mir-13a, mir-13b-1 and mir-13b-2* [2]. In this study, we found that overexpression of miR-2b-1 alone is sufficient to cause testicular bulging phenotype in *D. melanogaster*. In order to understand the genes and/or pathways regulated by the miR-2b-1 for the observed phenotype, miR-2b-1 overexpressing 3-5 days old adult male flies were dissected to obtain the testes for RNA extraction and sequencing. RNA sequencing was performed using Illumina Hiseq platform. Six paired-end raw reads were generated, 3 for control (*act5C-GAL4>OreR*) and 3 for miR-2b-1 overexpression (*act5C-GAL4>UAS-miR-2b-1*) respectively. The clean raw reads were mapped using RNA STAR and differential gene expression was performed using edgeR. Galaxy version 22.05 was used to perform these analyses. Differentially expressed genes along with their respective fold change and expression levels as count per million (CPM) are listed in Supplementary 1. The significantly differentially expressed genes are then classified according to the Gene ontology (GO) terms and these data are presented in the Figs. 1, 2 and 3.

## 3 Experimental Design, Materials and Methods

### 3.1 Fly husbandry

White (w1118) flies (BDSC#3605), UASp-miR-2b-1/TM3,Sb,Ser (BDSC#59852) and Act5C-GAL4/Cyo (BDSC#4414) were used in this study. Stock flies were maintained at 25˚C, 12 h light/dark cycle in a corn-based meal consists of 4% (w/v) corn starch, 5% (w/v) polenta, 10% (w/v) brown sugar, 0.7% (w/v) agar, 5% (w/v) yeast, 3% (v/v) nipagin and 0.5% (v/v) propionic acid. All crosses were made at 28˚C. F1s with genotype *act5C-GAL4>w1118* (control) and *act5C-GAL4>UAS-miR-2b-1* were collected upon eclosion and the testes of the adult flies aged between 3-5 days were dissected.

### 3.2 Total RNA extraction, library construction, and RNA-seq

Approximately 90-100 testes were pooled together for each replicate of RNA extraction using the combination of Trizol reagent (Invitrogen, USA) and RNeasy Mini Kit (Qiagen, Germany) as previously mentioned in Woo et al [3]. Total RNA was used for cDNA library construction following the protocol supplied with the Truseq™ RNA sample prep Kit (Illumina, San Diego, USA). Amplified cDNA fragments were sequenced by Illumina HiSeq^TM^ platform with 2 × 150bp. Raw data generated was trimmed and cleaned by removing low quality reads and removing the adaptor.

### 3.3 Differential expression analysis

Galaxy version 22.05 was used to perform differential gene expression [4]. Cleaned RNA-seq reads were aligned to the reference genome of *D. melanogaster* by using RNA STAR version 2.7.8a [2]. The genome file, Drosophila_melanogaster.BDGP6.87.gtf was downloaded from Ensembl. To measure gene expression counts featureCounts was used [3]. Differential gene expression was analyzed using edgeR [5]. FDR< 0.05 were set as the threshold for significantly differential expression genes.

### 3.4 GO classification and enrichment

DAVID online tool was used to identify significantly enriched GO terms (*P*-value<0.05) featuring biological process, cellular component, and molecular function [6].

## Ethics Statements

All animal handlings complied with guidelines set forth by the National Institutes of Health for the care and use of laboratory animals, and the protocol of this study followed the National Institutes of Health guide for the care and use of laboratory animals (NIH Publications No. 8023, revised 1978) and Guide for the Care and Use of Laboratory Animals: Table 4 8th Edition. Only adult male flies were used for data collection.

## CRediT authorship contribution statement

**Sharvin Manickam:** Conceptualization, Methodology, Software. **Shallinie Thangadurai:** Data curation, Writing – original draft. **Azali Azlan:** Conceptualization, Investigation, Software, Validation. **Zarina Amin:** Writing – review & editing, Resources. **Ghows Azzam:** Supervision, Writing – review & editing, Funding acquisition. **Mardani Abdul Halim:** Supervision, Resources, Writing – review & editing.

## Declaration of Competing Interest

The authors declare that they have no known competing financial interests or personal relationships that could have appeared to influence the work reported in this paper.

## Data Availability

RNA-seq analysis of Drosophila melanogaster adult testes overexpressing miR-2b-1 (Original Data) (NCBI).

## Appendix Supplementary materials

Image, application 1
